# A Robust Multi-Branch CNN-LSTM Architecture for Cross-Subject Motor Imagery Classification

**DOI:** 10.3390/s26113310

**Published:** 2026-05-23

**Authors:** Simone Zini, Federico Bidone, Paolo Napoletano

**Affiliations:** Department of Informatics, Systems and Communication, University of Milano-Bicocca, Viale Sarca 336, 20126 Milano, Italy; f.bidone1@campus.unimib.it

**Keywords:** BCI, motor imagery, deep learning, LSTM, EEG

## Abstract

Brain–computer interfaces (BCIs) based on motor imagery (MI) aim to convert electroencephalographic (EEG) activity into reliable device commands across users and recording setups. However, low signal-to-noise ratio and strong inter-subject variability still limit true “plug-and-play” deployment without lengthy calibration. To address these challenges, we propose a multi-branch convolutional long short-term memory (CNN-LSTM) architecture that jointly performs multi-scale temporal feature extraction and within-trial sequence modeling. The model employs four parallel 1D convolutional branches with distinct kernel sizes, each followed by an LSTM module and late fusion, combined with group normalization and supervision over sequences of sub-windows within each trial. We evaluate the approach on the EEG Motor Movement/Imagery (EEGMMI) dataset from PhysioNet under strictly subject-independent conditions, and on the ISLab-MI Dataset, a 32-channel wearable-EEG collection designed to assess cross-setup robustness. On EEGMMI, the network achieves up to 82.63% accuracy for binary left/right MI and 74.10% for a four-class task using 4 s trials under 5-fold cross-validation, outperforming an EEGNet-style baseline by 1–10% depending on class count and window length. Under a leave-one-subject-out protocol, the model attains 74.9% mean accuracy for a three-class MI task. Zero-shot transfer to ISLab-MI yields 64.60% and 63.02% accuracy in three- and four-class settings, respectively, while brief subject-specific fine-tuning using only 20% of each session improves performance to 81.38% and 73.48%. These findings show that combining multi-scale convolutional feature extraction with explicit sequence modeling and robust normalization yields accurate, data-efficient, and portable MI decoders suitable for practical BCI applications.

## 1. Introduction

Brain–computer interfaces (BCIs), which convert neural signals into device control, create real-world possibilities for rehabilitation and assistive mobility. Of the non-invasive options available, electroencephalography (EEG) is an appealing option due to its safety, portability, and high-resolution temporal information  [1]. When combined with modern artificial intelligence, in particular when using deep learning techniques, EEG provides the capability to perform end-to-end neural decoding through the use of discriminative features learned from the input data [2]. The combination of BCI technology and deep learning is key for applications that require users to express intent without producing residual muscle activity, i.e., operating a powered wheelchair.

Motor imagery (MI) provides a natural control paradigm for EEG BCIs. Imagining limb movements modulates oscillatory activity over sensorimotor cortices, with characteristic changes in the mu (about 8–12 Hz) and beta (about 13–30 Hz) bands. These sensorimotor rhythms exhibit event-related desynchronization and resynchronization that track imagined actions and their timing [3]. Because MI requires no overt movement, it is suitable for people with severe motor impairments and can be mapped to navigation primitives such as start, stop, and turns, or to higher level behaviors orchestrated by a shared controller [1,4].

While there has been significant advancement in the field of MI-based BCIs, specifically due to advancements in the application of deep learning techniques, MI-based BCIs still experience challenges in accurately decoding MI from EEG. Signals typically have a low signal-to-noise ratio, are non-stationary across trials and sessions, and are highly variable between subjects. Therefore, models that are trained on one subject will often fail when applied to another subject. Many current BCI systems continue to utilize a time-consuming calibration process that must be completed for each new user. To achieve “plug-and-play” capabilities, researchers need to develop methods that can identify subject invariant representations while maintaining the ability to detect the relevant spatial and temporal structure of MI. Recent research in cross-subject decoding views this challenge as a distribution shift problem and proposes domain generalization strategies to address it [5].

A large body of research has utilized both traditional signal processing methods and deep learning methods to meet these requirements. In early BCI systems, researchers utilized handcrafted features (such as band power and spatial filters) and used shallow classifiers. However, early systems were unable to handle the non-stationariness of the data and the complexity of multi-class classification problems. The development of compact convolutional neural networks, specifically EEGNet, demonstrated that depthwise and separable convolutions can learn spatial–spectral structure directly from unprocessed EEG and generalize well across different paradigms while maintaining low parameters [6].

More recent research has proposed architectures utilizing attention mechanisms to model longer-term temporal dependencies. The hybrid CNN-Transformer architecture utilizes the local inductive biases provided by convolution layers to provide spatial and spectral features, and the global context integration provided by self-attention layers to provide contextual information; in MI classification, these architectures have shown improvements over purely convolutional architectures, in addition to providing better interpretability through the use of attention maps [7]. However, much of the existing research continues to focus on either spatial or temporal aspects of the data, require subject-specific adaptation processes, and/or do not provide sufficient insight regarding how decisions are made using specific combinations of channels and time segments [2,5].

Taken together, recent advances in motor imagery decoding indicate that no single modeling strategy is sufficient to fully address the challenges of practical cross-subject BCIs. Multi-scale convolutional architectures such as TSception [8] and HS-CNN [9] improve sensitivity to EEG patterns evolving at different temporal resolutions, while recent transformer-based and hybrid sequence models, including EEG-Conformer [10], GRUGate-Trans [11], and t-CTrans [12], enhance contextual integration and long-range temporal modeling through attention and recurrent gating mechanisms. In parallel, domain adaptation and domain generalization approaches explicitly address inter-subject distribution shifts by promoting more invariant representations across users. Overall, these developments suggest that robust cross-subject decoding requires simultaneously handling multi-scale temporal structure, informative context aggregation, and heterogeneous subject distributions.

In this context, we target robust cross-subject MI decoding by combining multi-scale temporal feature extraction with explicit within-trial sequence modeling. Concretely, we:

- Introduce a multi-branch 1D CNN–LSTM architecture that captures complementary temporal scales and integrates evidence over sub-windows via late fusion;
- Adopt group normalization to improve training stability and reduce dependence on batch statistics in mixed-subject training;
- Evaluate in strictly subject-independent settings (5-fold cross-subject CV and LOSO) on large EEGMMI cohorts (105–109 subjects) with controlled windowing/sub-windowing;
- Quantify the benefit of rapid subject-specific transfer learning following Wang et al. [13], assessing both zero-shot and few-shot personalization.

## 2. Related Works

Classical pipelines for motor imagery (MI) EEG decoding relied on hand-crafted features and shallow classifiers. The common spatial pattern (CSP) algorithm was a cornerstone for two-class MI because it maximizes variance differences between classes, yet it typically requires supervised, subject-specific calibration to achieve reliable control [14]. Multi-subject learning strategies alleviate this burden by estimating spatial filters from groups of participants; for instance, Devlaminck et al. [15] showed that leveraging data from other users can reduce the amount of labeled trials needed for a new subject. In parallel, Riemannian geometry methods treat trial covariance matrices as points on the manifold of symmetric positive definite (SPD) matrices and use geodesic distances or tangent-space projections to obtain robust representations [16]. These approaches often outperform CSP and reduce calibration time in MI tasks [14], but they still depend on manually designed filter banks and shallow decision rules and, crucially, they struggle to capture the long-range temporal structure of non-stationary EEG.

The advent of deep convolutional neural networks (CNNs) has enabled end-to-end learning of spatial–spectral filters from raw EEG, enabling development of more complex and capable analytical tools. It has been demonstrated that shallow/deep ConvNets can either perform equally well or better than filter bank-based methods for MI Schirrmeister et al. [17] with added benefits including visualization of the learned features.

Lawhern et al. [6] proposed EEGNet, a compact architecture that employs depthwise and separable convolutions to disentangle spatial from temporal filtering, achieving competitive accuracy with few parameters and generalizing across multiple BCI paradigms. Building on these ideas, multi-scale temporal CNNs deploy parallel or hybrid kernel sizes to capture rhythms spanning alpha/beta bands; for example, HS-CNN integrates multiple temporal receptive fields within each layer and improves MI performance by aggregating information across scales [9]. Such models learn richer spatial–spectral features than classical pipelines, but their temporal receptive field remains bounded by kernel size and depth; without an explicit sequence module, they may under-exploit dependencies spread across sub-windows within a trial.

To address temporal integration, many works couple CNN front-ends with recurrent neural networks (RNNs). In these hybrid architectures, usually a CNN extracts features from short segments of EEG recordings and an RNN (e.g., LSTM/GRU) aggregates them over the 3–5 s MI trial. Zhang et al. [18] introduced a parallel CNN-RNN scheme for four-class MI that outperformed standalone CNNs, and Khademi et al. [19] showed that a transfer-learning-based CNN-LSTM markedly improves BCI Competition IV-2a performance after fine-tuning. Temporal convolutional networks (TCNs) with dilated convolutions provide a non-recurrent alternative to enlarge the temporal receptive field [9]. Overall, these hybrids consistently help when discriminative cues are distributed over time (e.g., early cue-locked responses vs. sustained ERD/ERS). However, their effectiveness depends on how segments are defined, which normalization is used to stabilize training across subjects, and how temporal features are fused.

Attention mechanisms and transformer-based models have been applied to MI EEG to weight informative cue/ERD–ERS portions of each trial and to model dependencies across sensorimotor channels. Representative examples include EEG-Conformer [10] and related local–global hybrids. Zhang et al. [7] proposed an approach which pairs a CNN local feature extractor with a transformer encoder that models global structure via multi-head self-attention; in MI classification, this design reported gains of a few percentage points over CNN baselines, alongside attention maps that highlight physiologically plausible channels and time ranges. Tao et al. [11] enhanced the standard Transformer by replacing residual connections with GRU-based gating mechanisms, applied to EEG sequences pre-processed through temporal and spatial 1D convolutions, to improve long-range dependency modeling for motor imagery and visual EEG classification. Xie et al. [12] designed a Transformer-based model that jointly exploits spatial (cross-channel) and temporal dependencies in raw EEG signals, demonstrating strong cross-subject generalization on motor imagery tasks from the PhysioNet dataset. More recently, Muna et al. [20] proposed SSTAF, combining a spectral and a spatial Transformer branch, fed with STFT-derived time-frequency features, to simultaneously attend to discriminative patterns across the spectral, spatial, and temporal domains for motor imagery classification. While attention improves modeling power and provides transparent saliency, transformer-heavy architectures can be data- and computation-hungry and require careful regularization to avoid overfitting noisy EEG [2].

Despite these advances, robust generalization to unseen subjects remains a major bottleneck for practical MI BCIs. Inter-subject variability affects both the spatial signatures of sensorimotor rhythms (due to anatomy, montage differences, and impedance) and the temporal profile of ERD/ERS within each trial (latency, duration, and strategy-dependent timing). As a result, models that emphasize a single temporal scale or that aggregate features without explicitly modeling within-trial dynamics may under-utilize informative time structure and degrade under subject shift. In addition, training stability can be sensitive to normalization choices when batches mix different subjects. For this reason, we focus on architectures that (i) capture complementary temporal scales, (ii) integrate evidence explicitly over a sequence of sub-windows, and (iii) employ normalization schemes that are less dependent on batch statistics. An initial attempt to resolve this type of problem consists of adopting unsupervised domain adaptation, which aligns the representations of the source and target subjects during training: adversarial approaches in the spirit of DANN add a gradient-reversal branch that encourages subject-invariant features and have improved subject-independent MI decoding [21]. Riemannian alignment (for example, Euclidean/whitening alignment of covariance or tangent-space centering) is complementary and forms a strong baseline for cross-subject MI [16]. Recent surveys emphasize that deep decoders without explicit alignment or normalization can underperform on new users and that robust normalization is crucial for cross-subject stability [2]. Beyond adaptation, domain generalization (DG) trains on multiple sources to learn invariances transferable to unseen users; teacher–student distillation, correlation alignment, and data-centric augmentations (noise, time-warping, mixup) have all been explored, often in combination, to mitigate inter-subject shift [2,5]. Despite encouraging progress, reported gains vary with datasets, windowing choices, and evaluation protocols, underscoring the need for carefully controlled comparisons. Even with domain adaptation or domain generalization, a residual “last-mile” gap often remains when moving to a new user or recording setup. In many cases, however, collecting a small amount of labeled data from the target user is feasible and can substantially improve reliability. Subject-specific transfer learning (SS-TL) is therefore a pragmatic strategy: a decoder is pretrained on a large multi-subject cohort and then quickly fine-tuned with a limited number of labeled trials from the new subject.

A common workflow pretrains a compact decoder on many subjects and then fine-tunes a small head or adapter using a few labeled trials from the new user. Wang et al. [13] showed that an EEGNet-based MI decoder can be efficiently adapted on a low-power edge device, with brief on-device updates yielding consistent gains without cloud retraining. Similar benefits were observed for CNN-LSTM hybrids [19]. SS-TL thus trades a short calibration session (minutes) for improved reliability, often the most practical option for deployment.

In summary, while prior work has made substantial progress in spatial filtering, temporal modeling, and subject-specific adaptation, robust generalization to unseen users remains challenging. This motivates architectures that jointly capture multi-scale temporal structure, explicitly model within-trial dynamics, and rely on normalization schemes that remain stable across heterogeneous subjects—objectives that guide the design of our proposed CNN–LSTM model.

## 3. Proposed Model

**Figure 1:**
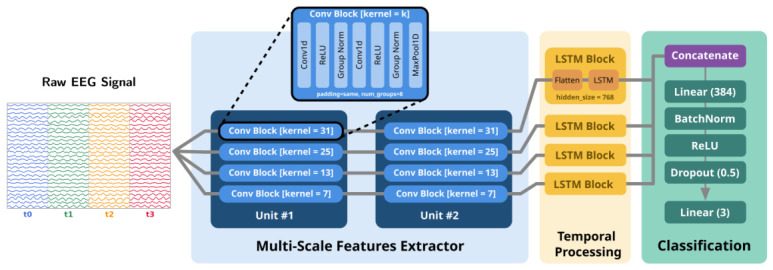
Schematic overview of the proposed architecture. Four temporal CNN branches with kernel sizes {31,25,13,7} extract multi-scale features and reduce the temporal resolution by a factor of four; each branch is followed by a single-layer LSTM (H=768). The LSTM outputs are concatenated and classified per time step by a shallow MLP, yielding logits of shape $\left(B,T,N_{\mathrm{classes}}\right)$.

Our architecture, illustrated in Figure 1, is a multi-branch CNN-LSTM designed to learn complementary temporal features at different receptive-field scales and to integrate them over the sequence of sub-windows that compose each event. The network expects as input a 4D tensor $X\in R^{B\times T\times C\times L}$, where *B* is the batch size, *T* the number of sub-windows in the event, *C* the number of input channels, and *L* the number of time samples per sub-window. Throughout this section we keep *C* generic, with the only constraint being that it must be divisible by eight due to the group normalization configuration described below.

The model is divided into multiple components: a first feature extractor part, followed by temporal feature analysis and classification. The first component is a parallel 1D-CNN feature extractor with $N_{\mathrm{branches}}=4$ branches operating in parallel on the same input. Branch b∈{1,…,4} uses a kernel size $k_{b}$ drawn from the ordered set {7,13,25,31}, so that each branch specializes to a distinct temporal scale. Given the common resampling rate of 160 Hz adopted in all experiments, the kernel sizes 7, 13, 25, 31 correspond to temporal receptive fields of approximately 44–194 ms. This range was chosen to capture short-term transient modulations of sensorimotor rhythms while preserving neighboring temporal resolutions across branches. Rather than targeting a single frequency band explicitly, the proposed design enables the network to learn complementary temporal patterns related to mu/beta desynchronization and rebound phenomena [22]. Within every branch, temporal processing is performed by stacking in series $D_{\mathrm{branch}}=2$ identical *ConvBlock*s. Each *ConvBlock* consists of two temporal convolutions that preserve the channel dimension followed by normalization, rectification, and downsampling along time: specifically, $\mathrm{Conv}1d(C\;\to \;C,\;k_{b},\;\mathrm{padding}=\mathrm{same})$, GroupNorm(8,C), ReLU, $\mathrm{Conv}1d(C\;\to \;C,\;k_{b},\;\mathrm{padding}=\mathrm{same})$, GroupNorm(8,C), ReLU, and finally MaxPool1d(kernel=2,stride=2). Convolutions use unit stride and are bias-enabled; “same” padding keeps the temporal length invariant inside each convolution so that the only temporal downsampling comes from the max pooling. Because two *ConvBlock*s are applied in sequence, the temporal length is reduced by a factor of four within each branch, whereas the channel count remains *C*. Implementation-wise, the tensor is first reshaped to (B·T,C,L) to process the *T* sub-windows independently; after the two blocks, each branch produces (B·T,C,L/4), which is then flattened to $\left(B,T,F_{\mathrm{branch}}\right)$ with $F_{\mathrm{branch}}=C\cdot \left(L/4\right)$. This flattening makes the subsequent recurrent module agnostic to the spatial arrangement of channels while retaining all information preserved by the convolutions.

Temporal processing across the *T* sub-windows is performed per branch by a single-layer LSTM configured with batch_first=True, hidden size $H_{\mathrm{LSTM}}=768$, input size $F_{\mathrm{branch}}$, and default PyTorch (v 2.7.1) gates and activations (no recurrent dropout, unidirectional). Feeding $\left(B,T,F_{\mathrm{branch}}\right)$ yields a sequence of hidden states $\left(B,T,H_{\mathrm{LSTM}}\right)$ per branch. The four sequences are then concatenated along the feature axis, resulting in $\left(B,T,4H_{\mathrm{LSTM}}\right)$. This late fusion preserves the per time step alignment across branches while maximizing the representational capacity; in practice, it is equivalent to running four independent LSTMs and stacking their outputs channel-wise.

Classification is performed at the time-step level by a lightweight MLP applied identically to each row of the fused sequence. Concretely, the tensor $\left(B,T,4H_{\mathrm{LSTM}}\right)$ is reshaped to $\left(B\;\cdot \;T,4H_{\mathrm{LSTM}}\right)$, passed through a fully connected layer to 384 units, followed by BatchNorm1d(384), ReLU, and Dropout(p=0.5), and finally projected to $N_{\mathrm{classes}}$ logits by a second fully connected layer. The output is reshaped back to $\left(B,T,N_{\mathrm{classes}}\right)$. We deliberately keep the classifier shallow to concentrate capacity in the recurrent integration, which empirically carries the bulk of temporal modeling. During training, a standard cross-entropy loss is computed at every time step using the event label repeated *T* times; this objective encourages consistent predictions across the sub-windows of the same event without imposing hard constraints on the internal dynamics. At inference time, if a single decision per event is desired, the model’s per-step logits can be averaged across the *T* positions (or, equivalently, a majority vote can be taken on the per-step argmax), but such aggregation is external to the architecture and does not affect training. The overall architecture, in the configuration with four branches and the subsequent temporal correlation step and classification, presents a computational complexity corresponding with 1.197 GFLOPs.

The code is available at: https://github.com/unimib-islab/EEG-BCI-Cross-Subject-Motor-Imagery (accessed on 20 May 2026).

## 4. Datasets

We evaluate the proposed model on a large public EEG motor imagery dataset and on a smaller ISLab-MI dataset acquired with a wearable system, in order to assess both cross-subject robustness and portability across recording setups.

### 4.1. PhysioNet EEG Motor Movement/Imagery Dataset

We evaluate the proposed model on the EEG Motor Movement/Imagery (EEGMMI) dataset hosted on PhysioNet [23]. EEGMMI includes recordings from 109 subjects who performed 14 experimental runs spanning motor execution and motor imagery (MI) tasks. Signals were acquired from 64 scalp electrodes arranged according to the international 10 –10 system. Following common practice, we focus on four MI classes (left fist, right fist, both fists, both feet) and a resting state (0). Unless otherwise specified, our subject pool includes 105 participants (excluding subjects 89, 92, 100, and 104 due to data inconsistencies). Comparisons that require all participants are reported on the full 109-subject set for completeness. Each recording is: (i) notch-filtered at 60 Hz to suppress power-line noise; (ii) resampled to a uniform 160 Hz; and (iii) z-score normalized per channel using statistics computed strictly on the training subjects within each cross-validation fold. Those statistics are then applied to both training and test sets for the fold. This step proved important for training stability and cross-subject generalization.

**Figure 2:**
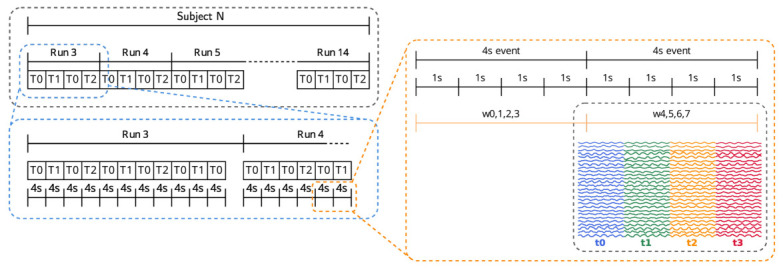
EEGMMI segmentation into macro-windows and sub-windows. Each macro-window contains a labeled event performed by the subject.

Each labeled event (macro-window) lasts 4 s and is partitioned into a sequence of *T* non-overlapping sub-windows of duration $w_{\sec}$ seconds, yielding $T=4/w_{\sec}$. With C=64 channels and a sampling rate of 160 Hz, each sub-window contains $L=160\cdot w_{\sec}$ time samples. The resulting input tensor for a batch of *B* macro-windows is $X\in R^{B\times T\times C\times L}$, matching the model contract introduced in Section 3. An illustration of the segmentation is provided in Figure 2.

### 4.2. The ISLab-MI Dataset

In order to test model generalization capabilities, we collected a motor imagery (MI) dataset, called ISLab-MI Dataset, with a wearable g.Nautilus PRO system. Recordings were performed on five healthy adult volunteers (20–40 years) recruited from the university community. Each participant completed one experimental visit in a quiet laboratory room, seated comfortably in front of a monitor and instructed to minimize head and body movements and to avoid eye blinks during the active phases. The visit was structured as two consecutive EEG sessions separated by a short rest break, yielding two internally consistent datasets per subject; across participants and visits this resulted in ten recording sessions in total. The amplifier was used with the full 32-electrode wet cap arranged according to an extended 10–20 layout, plus one auxiliary channel, for a total of 33 recorded channels. Raw EEG was acquired with g.Recorder at 500 Hz, 24-bit resolution and an input range of ±187.5 mV, so as to capture small-amplitude oscillatory activity while preserving headroom for artifacts. Event markers were delivered through the digital inputs of the base station to timestamp the onset and duration of each trial.

The MI protocol was based on a small set of discrete imagined movements designed for powered-wheelchair control. Each trial followed a structured sequence of phases: a *fixation* period in which the subject focused on a central cross; a *cue* phase in which a visual stimulus indicated the upcoming task; a *perform* phase during which the subject continuously imagined the required movement; and a *blank* period where the subject relaxed. The four MI classes corresponded to opening and closing the right hand, opening and closing the left hand, opening and closing both hands, and dorsiflexion of both feet, respectively. A no-movement baseline was also recorded and later treated as a separate rest class (0). Within each session, stimuli were presented in pseudo-random order with an equal number of repetitions per class so that all commands were equally represented.

To align training and inference with the model presented in Section 3, we converted all recordings to a uniform 32-channel montage (10 –10 subset) and a sampling rate of 160 Hz. The 32 channels were derived from the original 64-channel EEG montage by selecting a consistent subset of electrodes within the 10–10/10–20 system, so that all sessions shared the same spatial layout. In practice, we chose one electrode from each pair of closely spaced 10–10 sites and kept the main midline positions (Fpz, Fz, Cz, Pz, Oz). This produced a balanced set of frontal, central, parietal, temporal, and occipital channels, preserving the overall structure of the original montage while ensuring a uniform 32-channel configuration. Signal conversion and preprocessing followed a fixed pipeline: a mild high-pass at 0.2 Hz at the native sampling rate, polyphase resampling to 160 Hz, a 50 Hz notch (EU mains), and a 0.5–40 Hz band-pass. Signals were scaled to microvolts and channels were reordered to the 32-target layout used throughout this work. Events were reconstructed from the trigger channel using the “PERFORM” schedule adopted in our protocol. Each occurrence of the *perform* cue was mapped to one of four labels: left hand (L), right hand (R), both hands (F), or both feet (B), while “blank” segments were split into two 4 s rest windows (label 0). The final archive for each session follows a compact HDF5 layout containing raw data, an event table with onset, duration, and label, and a convenience group with per channel μ/σ computed once for analysis reproducibility.

Unless stated otherwise, we adopt the same temporal framing used for EEGMMI: each labeled event is a 4 s macro-window decomposed into T=4 non-overlapping sub-windows of 1 s (L=160 samples at 160 Hz), yielding inputs $X\in R^{B\times T\times C\times L}$ with C=32. For zero-shot evaluation, we standardize channels using the μ/σ statistics associated with the pre-trained EEGMMI models, ensuring strict consistency between training and deployment. We report three classification settings that mirror our public-data experiments: L/R/0 (3-class), L/R/0/F (4-class), and L/R/0/F/B (5-class).

### 4.3. Experimental Setup

We employ two distinct training and evaluation setups: one for cross-subject modeling on the public EEGMMI dataset and one for transfer learning and personalization on the ISLab-MI dataset.

#### 4.3.1. Training on EEGMMI

Unless otherwise noted, results are obtained with 5-fold subject-independent cross-validation on 105 subjects. In each split, one fold is held out for testing and the remaining k−1 folds are used for training. The training is then further split with a 90−10 split to obtain a validation set for each fold. Models are trained for up to 40 epochs with AdamW (learning rate $2.89\times 10^{-4}$, weight decay $5.82\times 10^{-4}$) and batch size 16. At the end of every epoch we evaluate accuracy and loss on the validation fold. Early stopping halts training if validation accuracy does not improve for 10 consecutive epochs, and the score reported for the fold is achieved using the model weights at the stopping epoch. Cross-entropy loss is computed over the predictions of all sub-windows.

#### 4.3.2. Pre-Training and Adaptation to the ISLab-MI Dataset

We retrained the proposed CNN-LSTM on EEGMMI restricted to the same 32-channel subset and imagery runs, using a leave-one-subject-out (LOSO) protocol over all 109 subjects. For each held-out subject we tracked the best epoch; the fixed training horizon for the final weights was set to the median of these best epochs, computed per task: eight epochs for the five-class configuration and seven epochs for the three- and four-class configurations. We then trained on the full 109-subject cohort at the corresponding horizon to obtain the final zero-shot models used below.

We evaluated both zero-shot transfer and rapid subject-specific adaptation on our dataset. For adaptation, we adopted a simple and reproducible split: the first 20% of events in each session were used for fine-tuning and the remaining 80% for testing. Fine-tuning ran for 10 epochs with Adam (classifier head learning rate $5\times 10^{-4}$, backbone $1\times 10^{-4}$), computing the loss on the per event decision obtained by averaging the model’s per sub-window logits. This protocol matches the SS-TL spirit of prior work while keeping the amount of subject data and training time minimal.

## 5. Results and Discussion

### 5.1. Comparison with State-of-the-Art

Since the different works adopt setups that are not directly comparable, we present the results in separate sections, maintaining the original evaluation conditions for each.We report here the main quantitative results obtained on the public EEGMMI dataset and on our ISLab-MI dataset, as well as the impact of SS-TL, in comparison with the selected state-of-the-art approaches. More specifically we compared the proposed solution with the approaches by Wang et al. [13], Ghimire and Sekeroglu [24], GRUGate-Trans [11], t-CTrans [12] and SSTAF Transformer [20].

Table 1 shows the comparison between our model and the EEGNet-based approach by Wang et al. [13] across two-, three-, and four-class MI settings and multiple window sizes. Our network consistently outperforms the baseline across comparable conditions. With 2 s and 1 s sub-windows we observe gains of roughly 2–10 percentage points depending on the number of classes, while 4 s windows provide our strongest absolute accuracies.

Inspired by the protocol of Wang et al. [13], we evaluated the model’s ability to perform transfer learning from the general cross-subject setting to the subject-specific case. The adopted protocol is as follows: we perform a 5-fold CV between subjects and, for each fold, we select the weights with the best accuracy on the test subjects. For each subject in the test set, we apply a further intra-subject subdivision into four folds, and perform a fine-tuning of the model in CV, obtaining four accuracy values. The average of the four values constitutes the accuracy of the single subject, the average of subjects defines the accuracy of the fold, and finally the average of the five fold represents the final performance of our SS-TL method.

We apply the SS-TL protocol on top of our cross-subject model. Table 2 summarizes the mean accuracies and absolute gains for the 2 s and 4 s window conditions across different class counts. These results confirm that the proposed architecture serves as a strong subject-agnostic backbone that can be efficiently personalized with limited subject-specific data.

To align with Ghimire and Sekeroglu [24], who report on all 109 subjects with a fixed 80/20 split, we also provide results in their setting. As shown in Table 3, our 5-fold average on 109 subjects exceeds their split-based accuracy, and the accuracy of our best fold is substantially higher.

On the stringent LOSO protocol for the three-class L/R/0 task (4 s window), our model attains respectively 74.5% and 74.9% with 105 and 103 subjects. In Table 4, we reported the results of GRUGate-Trans [11], t-CTrans [12] and SSTAF Transformer [20]. For each model we reported the LOSO setup with the actual number of subjects used by the authors in the original work. The numbers are taken from [20]. Even if the numbers are partially comparable, it is possible to notice the gain of the proposed model with respect to the first two models, while showing slightly lower results with respect to the more recent transformer architecture.

**Figure 3:**
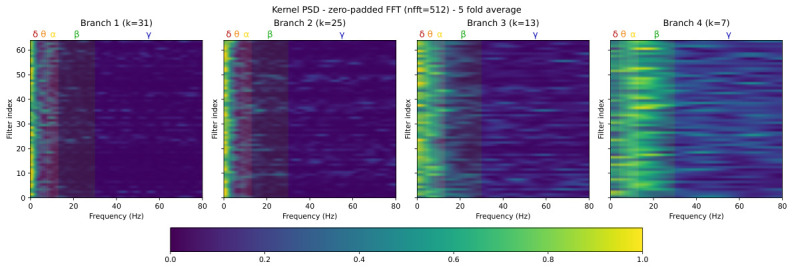
Power spectral density (PSD) of the learned first-layer convolutional kernels for the four temporal branches averaged across 5 folds. The redesigned kernel sizes, 31, 25, 13, 7, exhibit partially distinct spectral sensitivities, supporting complementary temporal processing across neighboring EEG frequency ranges.

To investigate whether the different temporal branches learn complementary representations, we analyzed the frequency response of the first-layer convolutional kernels after training. Figure 3 reports the power spectral density (PSD) of the learned filters averaged across folds for each branch. The kernel configuration 31, 25, 13, 7 corresponds to temporal receptive fields ranging from approximately 44 ms to 194 ms at the adopted sampling frequency of 160 Hz. These scales were selected to capture heterogeneous temporal dynamics associated with motor imagery EEG, including both smoother oscillatory components and faster transient variations. The spectral analysis reveals that the branches do not converge to identical responses. Larger kernels exhibit stronger emphasis on low-frequency activity, whereas smaller kernels retain broader spectral sensitivity extending toward higher-frequency components. Rather than enforcing strict one-branch/one-band specialization, the proposed architecture appears to learn complementary temporal filtering behaviors across neighboring EEG frequency ranges, including the physiologically relevant mu and beta bands.

### 5.2. Evaluation on the ISLab-MI Dataset

**Figure 4:**
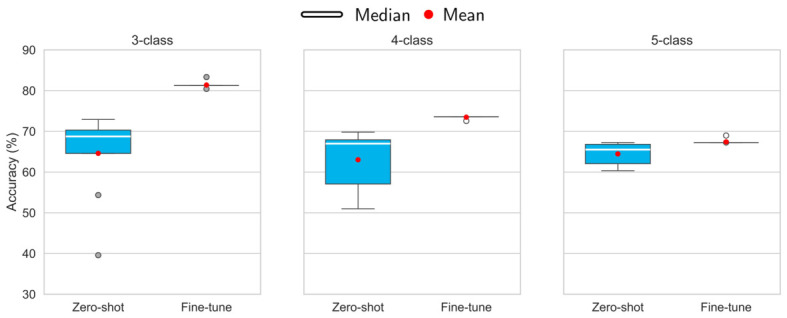
Accuracy distributions across zero-shot (ZS) and fine-tuned (FT) models for 3-, 4-, and 5-class classification tasks. Each boxplot summarizes subject and session level variability, with the median indicated by the horizontal line inside each box and the mean highlighted by a red dot marker. Fine-tuning consistently leads to higher accuracy and lower variability compared to the zero-shot condition across all classification settings.

Alongside the analysis on cross-subject performance on EEGMMI, here we report an examination on model capability to adapt to new datasets and a quantifications of the benefits from rapid subject-specific adaptation. Across the ten sessions, zero-shot performance was competitive, and fine-tuning consistently improved accuracy. In the three-class L/R/0 setting, mean accuracy increased from 64.60% ± 10.33 (zero-shot) to 81.83% ± 0.74 after fine-tuning. In the four-class L/R/0/F setting, accuracy rose from 63.02% ± 7.23 to 73.48% ± 0.33. In the more challenging five-class L/R/0/F/B setting, we observed a smaller but still positive gain from 64.46% ± 2.63 to 67.42% ± 0.54. Per-session results, including zero-shot baselines, best post-fine-tuning accuracies, and absolute improvements in percentage points, are reported in Table 5. The average accuracies, computed over all subjects and sessions, are reported in the final row of Table 5 and in the boxplot depicted in Figure 4. Taken together, these findings indicate that the proposed architecture, when pre-trained on a large public cohort, transfers robustly to recordings acquired with different hardware and benefits from short, session-specific calibration without requiring lengthy data collection.

## 6. Conclusions

Motor imagery BCIs promise hands-free control for assistive mobility, yet practical deployment is still limited by the low signal-to-noise ratio of EEG and, most critically, by inter-subject variability that breaks models trained on previous users. In this work, we addressed this bottleneck by designing a subject-robust decoder that explicitly targets the cross-subject variability that typically disrupts MI-BCI performance.

We introduced a multi-branch CNN-LSTM model for cross-subject motor imagery classification. The proposed architecture combines (i) multi-scale temporal feature extraction through parallel 1D convolutional branches and (ii) within-trial sequence modeling by per-branch LSTMs with late fusion. Together with group normalization and time-step supervision over sub-windows, this design stabilizes training in mixed-subject batches and encourages consistent predictions across the temporal evolution of each event. On the large EEGMMI cohort and under strictly subject-independent protocols, the proposed model consistently outperformed an EEGNet-style compact baseline across binary and multi-class settings, achieving high accuracy with 4 s trials and retaining strong performance even with shorter sub-windows. Under the more stringent LOSO evaluation, the model maintained competitive generalization and improved over recent transformer-based reports.

We also tested portability beyond the source dataset. When we transferred zero-shot to recordings acquired with different hardware and a different electrode montage, the EEGMMI-pre-trained model provided competitive accuracy, and brief subject-specific fine-tuning using only a small fraction of each session further closed the remaining gap. These results support a pragmatic deployment pathway: a strong cross-subject backbone for plug-and-play initialization, complemented by a short calibration stage that yields reliable control without lengthy data collection.

The encouraging zero-shot transfer results obtained on the ISLab-MI dataset suggest that the proposed architecture already captures partially transferable representations despite not relying on explicit domain adaptation objectives. A promising future direction consists of integrating the proposed backbone with domain-adversarial or invariant-learning strategies to further improve robustness across unseen subjects and acquisition setups.

Although the present analysis supports the relevance of the multi-branch temporal design, a fully exhaustive component-wise ablation remains an important direction for future work. In particular, replacing the LSTM with alternative temporal aggregation modules, such as temporal convolution, attention pooling, or transformer blocks, would allow for a more systematic assessment of the trade-off between sequence modeling capacity, computational cost, and cross-subject robustness. Similarly, further comparisons among normalization strategies could better quantify their impact under heterogeneous subject-independent training.

## Figures and Tables

**Table 1:** Classification accuracy (%) on 105 subjects with 5-fold subject-independent cross-validation. Comparison with Wang et al. [13]. Results from Wang et al. have been obtained by retraining their approach using the officially released code.

Classes	Window	Wang et al. [13]	Ours
	4 s	82.29% ± 2.00	82.63% ± 2.13
L/R (2)	2 s	81.20% ± 2.26	80.88% ± 3.10
	1 s	80.23% ± 2.19	81.45% ± 2.13
Average		81.24% ± 1.03	81.65% ± 0.41
	4 s	74.20% ± 2.98	74.10% ± 1.61
L/R/0 (3)	2 s	73.44% ± 1.93	73.50% ± 2.02
	1 s	72.32% ± 2.88	73.43% ± 1.73
Average		73.32% ± 0.95	73.68% ± 0.37
	4 s	65.26% ± 2.50	72.46% ± 1.67
L/R/0/F (4)	2 s	63.89% ± 2.74	73.19% ± 1.58
	1 s	61.81% ± 4.44	73.54% ± 1.88
Average		63.65% ± 1.74	73.06% ± 0.55
Avg. among	2 s	73.92% ± 8.52	76.40% ± 5.46
same window	3 s	72.84% ± 8.67	75.86% ± 4.35
length	4 s	71.45% ± 9.24	76.14% ± 4.60

**Table 2:** Subject-specific transfer learning (SS-TL) on EEGMMI. Results are computed on 105 subjects with 5-fold subject-independent cross-validation. Results from Wang et al. have been obtained by retraining their approach using the officially released code.

Window	Classes	Wang et al. [13]	Ours
	2-class	84.15% ± 15.71	84.89% ± 12.57
4 s	3-class	76.47% ± 15.14	78.58% ± 11.96
	4-class	67.31% ± 15.22	76.76% ± 11.05
	2-class	82.89% ± 16.24	82.86% ± 1.37
2 s	3-class	76.37% ± 13.31	78.52% ± 11.96
	4-class	66.54% ± 13.61	78.45% ± 11.03

**Table 3:** Comparison with Ghimire and Sekeroglu [24] on 109 subjects (4 s window).

Task	Validation	Ghimire et al. [24]	Ours
	Split (80/20)	82.79%	n/a
L/R (2-class)	5-fold CV	n/a	82.83% ± 2.39
	Best fold	n/a	87.20%
	Split (80/20)	69.08%	n/a
L/R/F (3-class)	5-fold CV	n/a	73.72% ± 1.71
	Best fold	n/a	76.58%

**Table 4:** LOSO accuracy (%) on the 3-class L/R/0 task (4 s window), as done by [20]. The results with the * symbol have been taken from [20].

Model	# of Subjects	LOSO Accuracy
GRUGate-Trans [11]	109	61.9% *
t-CTrans [12]	105	68.5% *
SSTAF [20]	103	76.8% *
Ours	105	74.5%
	103	74.9%

**Table 5:** Transfer learning on the ISLab-MI dataset: the accuracies for the 3-, 4- and 5-class classification tasks in the zero-shot and fine-tuned versions are reported for each subject’s session. The last row reports the average accuracy and standard deviation for the zero-shot and fine-tuned versions across all subjects.

		3-Class		4-Class		5-Class	
Subject	Session	Zero-Shot	Fine-Tune	Zero-Shot	Fine-Tune	Zero-Shot	Fine-Tune
1	1	64.58%	83.33%	67.92%	73.58%	62.07%	67.24%
	2	68.75%	81.25%	64.15%	73.58%	67.24%	67.24%
2	1	68.75%	81.25%	66.04%	73.58%	60.34%	68.97%
	2	39.58%	81.25%	52.83%	73.58%	67.24%	67.24%
3	1	64.58%	81.25%	54.72%	73.58%	62.07%	67.24%
	2	72.92%	81.25%	67.92%	73.58%	67.24%	67.24%
4	1	54.35%	80.43%	50.98%	72.55%	61.82%	67.27%
	2	70.83%	81.25%	67.92%	73.58%	65.52%	67.24%
5	1	68.75%	81.25%	69.81%	73.58%	65.52%	67.24%
	2	72.92%	81.25%	67.92%	73.58%	65.52%	67.24%
avg		64.60%	81.38%	63.02%	73.48%	64.46%	67.42%
std		10.327	0.734	7.230	0.328	2.623	0.544

## Data Availability

The public dataset supporting the results of this article is the EEG Motor Movement/Imagery (EEGMMI) dataset hosted on PhysioNet, and the authors confirm that the dataset is indicated in the reference list. The ISLab-MI dataset, collected by the authors, is available at https://github.com/unimib-islab/EEG-BCI-Cross-Subject-Motor-Imagery (accessed on 20 May 2026).
